# Adolescent chronic fatigue syndrome; a follow-up study displays concurrent improvement of circulatory abnormalities and clinical symptoms

**DOI:** 10.1186/1751-0759-6-10

**Published:** 2012-03-21

**Authors:** Dag Sulheim, Harald Hurum, Ingrid B Helland, Erik Thaulow, Vegard Bruun Wyller

**Affiliations:** 1 Department of Paediatric Medicine, Oslo University Hospital, Rikshospitalet, Oslo, Norway; 2 Department of Paediatrics, Innlandet Hospital Trust, Lillehammer, Norway; 3 Department of Paediatrics, Østfold Hospital Trust, Fredrikstad, Norway; 4 Department of Paediatric Medicine, Oslo University Hospital, Rikshospitalet N-0027 Oslo, Norway

**Keywords:** Chronic fatigue syndrome, Fatigue severity scale, Autonomic symptom profile, Cardiovascular autonomic control, Adolescents

## Abstract

**Background:**

The pathophysiology of chronic fatigue syndrome (CFS) in adolescents is unknown, and the clinical course and prognosis is still questioned. Recent research indicates that abnormalities of autonomic cardiovascular control may play an important role. The aim of this research project was to perform a follow-up study of adolescents with chronic fatigue syndrome, focusing on clinical symptoms and autonomic cardiovascular control.

**Methods:**

47 adolescents (12-18 years old) with CFS were recruited from the outpatient clinic at the Department of Pediatrics, Oslo University Hospital. In a primary visit and a follow-up visit (3-17 months later), we evaluated: a) a wide range of complaints and symptoms and b) cardiovascular variables at baseline and during a 20° head-up tilt-test (HUT).

**Results:**

At the second visit, patients reported significant improvement regarding functional impairments, fatigue severity, muscular pain, concentration problems, post-exertional malaise and the problem of non-relieving rest. Also, at the second visit, baseline heart rate (HR), blood pressure, total peripheral resistance index (TPRI) and LF/HF (low-frequency:high-frequency heart rate variability ratio, an index of sinus node sympathovagal balance derived from spectral analyses of heart rate) were significant lower, and the increases in HR, mean blood pressure (MBP), diastolic blood pressure (DBP) and TPRI during tilt were significantly less pronounced as compared to the first visit. There was a significant correlation between changes in autonomic symptom score, fatigue severity score and functional impairment score from the first to the second visit.

**Conclusions:**

The majority of adolescents with CFS experienced an improvement over time in functional impairment, self-reported fatigue and additional symptoms, and a concurrent improvement of autonomic cardiovascular control. A possible connection between clinical symptoms and abnormal autonomic control in CFS might represent a focus for further research.

## 

Chronic fatigue syndrome (CFS) is a disabling condition, seriously affecting school-attendance and social activities [1]. The prevalence among 8-17 year olds has been reported as high as 1.3% [2]; thus, CFS constitutes a substantial health problem in adolescence.

Patients with CFS frequently report complaints like nausea, lightheadedness and photophobia, symptoms indicative of disturbances in autonomic nervous activity. Experimental studies have demonstrated distinct abnormalities in cardiovascular autonomic control [3-5]. Previous reports from our institution have documented higher blood pressure, heart rate and LF/HF at rest among adolescent CFS patients as compared to healthy controls, and a stronger increase of these variables upon orthostatic stress [6-9]. Similarly, ambulatory measurements of blood pressure and heart rate indicate higher nocturnal values among CFS patients as compared to controls [10]. Taken together, these studies suggest that abnormal autonomic nervous activity might constitute an important aspect of CFS pathophysiology [11,12].

Several reports indicate a complete or near complete recovery in one third of patients; a similar number experienced no improvement [13-16]. However, the course of the broad spectrum of symptoms and complaints in adolescent CFS is poorly investigated; this is particularly true for symptoms of disturbances in autonomic nervous system activity. Likewise, to our knowledge, there is no study exploring the change in cardiovascular autonomic control over time.

The aim of this study was to explore the parallel course of clinical symptoms and abnormalities in autonomic cardiovascular control in adolescent CFS patients. We hypothesized an improvement of all variables with time, and a positive correlation between changes in fatigue, haemodynamics and functional impairments.

## Patients and methods

### Patients

During the study period from August 2007 to April 2009, adolescent CFS patients aged 12-18 years were recruited from the Paediatric outpatient clinic, Oslo University Hospital, Rikshospitalet, Norway, which serves as a national referral center for children and adolescents with unexplained chronic fatigue. Prior to referral, they had been tentatively given the diagnosis CFS at local hospitals.

Different case definitions of CFS exist. The frequently used definition from the International Chronic Fatigue Syndrome Study Group (commonly referred to as the Fukuda-definition) requires at least six months of unexplained chronic or relapsing fatigue of new onset, severely affecting daily activities, as well as four or more of eight specific accompanying symptoms (headache, muscle pain, joint pain, sore throat, tender lymph nodes, impaired memory or concentration, unrefreshing sleep, and malaise after exertion) [17]. The validity of this definition has been questioned in adults [18] and children [19]. Therefore, in this study, the inclusion criterion was three or more consecutive months of unexplained disabling fatigue worsened by physical or mental exertion. No other accompanying symptoms were required for inclusion. This approach is in line with the clinical recommendations from The Royal College of Paediatrics and Child Health [1] and the National Institute for Health and Clinical Excellence [20], and was also proven feasible in previous studies from our group [6-10]

Generally, no kind of treatment had been established prior to referral. At our institution, other disease states that might explain their present symptoms, such as autoimmune, endocrine, neurologic or psychiatric disorders (including depression), were ruled out by a thorough and standardized set of investigations. Approximately 50% of the referred patients were diagnosed as having CFS according to the criterion specified above, and were thus eligible for this study. All patients were given advice about activity adjustment and cognitive behavioral therapy in line with internationally accepted guidelines [20], whereas the establishment of treatment and rehabilitation programs was entrusted to local health service providers.

### Study protocol

CFS patients were invited to an experimental session (consisting of head-up tilt-test (HUT) and a questionnaire) at our institution at the time of their first clinical visit, and summoned for a second session 3-17 months later. One week prior to the experiments, all participants were instructed not to drink beverages containing alcohol or caffeine, not to take any drugs, and not to use tobacco products. Written, informed consent was obtained from all participants and their parents. The study was approved by the Regional committee for ethics in medical research.

### Questionnaire

Items from the Autonomic Symptom Profile (ASP) [21] and the Fatigue Severity Scale (FSS) [22], which are validated instruments for assessing autonomic dysfunction and fatigue, respectively, were translated into Norwegian by one of the authors (VBW) and slightly modified in order to fit our particular age group. The modified ASP consists of 22 items addressing orthostatic intolerance, vasomotor symptoms, sudomotor symptoms, gastrointestinal function, eye reflexes and sleep problems; the response alternatives are partly dichotomous ("yes" and "no") and partly 1-5 Likert scale. The modified FSS consists of 7 items having a 1-5 Likert scale. In addition to these instruments, we added items addressing common CFS symptoms (such as problems of concentration and memory) and functional impairments (such as school absenteeism), all answered on 1-5 Likert scales. The questionnaire has proven feasible in previous studies from our institution [7,8].

Patients were also interviewed regarding therapeutic interventions during the period between the first and second experimental session.

### Head-up tilt-test (HUT)

HUT was undertaken according to a revised protocol established by Wyller et al. [8]. The feasibility of this protocol for studying adolescent CFS patients has been demonstrated in several previous studies [6,8]. In particular, the low tilt angle (20°) does not normally precipitate syncope, which is otherwise a common problem among adolescents being subjected to stronger orthostatic challenges [23]. Still, a 20° head-up tilt is sufficient to demonstrate hemodynamic alterations among CFS patients compared to healthy controls.

HUT was performed at daytime between 8 a.m. and 3 p.m. under calm conditions, dimmed lights and a stable room- temperature of 23-25°C. Patients were attached to the Task Force Monitor^® ^(Model 3040i, CNSystems Medizintechnic, Graz, Austria); a combined hardware and software device for noninvasive continuous recording of cardiovascular variables [24]. They were positioned horizontally on a tilt-table with foot-board support (Model 900-00, CNS-systems Medizintechnik, Graz, Austria), with a safety strap over the waist. After 5 minutes of baseline recordings, they were head-up tilted 20° for 15 minutes, followed by another 5 minute epoch in a horizontal position. Subjects did not speak and were not spoken to.

Instantaneous heart rate (HR) was obtained from the R-R interval of the electrocardiogram. Photoplethysmography on the right middle finger was used to obtain a non-invasive, continuous recording of arterial blood pressure. This method correlates satisfactorily with invasive pressure measurements [25], and has been validated in adolescents and children [26]. Impedance cardiography was used to obtain a continuous recording of the temporal derivate of the transthoracic impedance (dZ/dt) [27].

All recorded signals were on-line transferred to the built-in recording computer of the Task Force Monitor^®^, running software for real-time data acquisition. Beat-to-beat mean arterial blood pressure (MBP) was calculated by numerical integration of the recorded instantaneous blood pressure signal. Beat-to-beat stroke volume (SV) was calculated from the impedance signal [24,28]. This method has been validated both in adults [29] and children [30]. The RR-interval from the heart rate recording was subjected to spectral analysis using an adaptive autoregressive algorithm, creating a time-varying spectrum [31]. Spectral power densities (absolute values) were calculated in the low-frequency (LF) band (0.04-0.15 Hz) and the high-frequency (HF) band (0.15-0.4 Hz); we also calculated the LF/HF. These indices are measures of autonomic heart rate control; the LF/HF is a measure of "sympathovagal balance", where higher values indicate enhanced sympathetic activity [32].

### Data analysis

Questionnaire data were exported to Microsoft Excel for further analyses. The answers to all ASP items were dichotomized and given the value 0 (symptom not/rarely present) or 1 (symptom often/always present). An Autonomic symptom score was then defined as the sum across all ASP items, having a total range from 0 to 22. A Fatigue severity score was created by taking the arithmetical mean across all 7 items from FSS; total range is from 0 to 5. Likewise, a Functional impairment score was defined as the arithmetical mean across three items addressing, respectively, school absenteeism, leisure activities and being with friends on a 1-5 Likert scale; thus, total range is from 1 to 5.

The HUT data from the Task Force Monitor was exported to Microsoft Excel for further analysis. From each experimental run of HUT, we calculated the median of all variables in two epochs: From 270 to 30 seconds prior to tilt (Baseline) and from 300 to 540 seconds after being tilted (Tilt). We also computed delta values (Tilt--Baseline). Beat-to-beat total peripheral resistance index (TPRI) was calculated as MBP divided by the product of SV and HR, and then indexed against body surface area (BSA) [33]. This method of analyzing results from HUT has successfully been used in previous projects from our institution [6,8].

Statistical analyses were carried out using SPSS statistical software (SPSS Inc. Chicago, IL, USA). Inspection of plots displayed lack of normal distribution for several variables; consequently, we have used non-parametric statistics: Wilcoxon signed rank test was applied for comparison of results from the two visits, whereas Kendall rank correlation was used to explore the relationship between changes in variables from the first to the second visit. A p-value < 0.05 was considered statistically significant.

## Results

During the recruiting period, 47 patients were included in the study. Six patients refused a second visit and were lost to follow-up. These patients (3 females and 3 males) were principally equal to the included patients regarding age, height and weight, fatigue severity score, and baseline HR and MBP. Of the remaining 41, one was excluded due to reevaluation of the diagnosis and two did not answer the questionnaire, leaving 38 patients (30 females, 8 males; 27 fulfilling the Fukuda definition of CFS) for the final analyses. Demographic details are given in Table 1.

**Table 1:** Subject characteristics

	*First visit*	*Second visit*
Total number, n	38	38

Participants satisfying the Fukuda definition of CFS, n (%)	27	
	(71)	

Age (years), median (range)	15	16
	(12-18)	(13-20)

Length (cm), median (range)	165	166
	(146-187)	(147-189)

Weight (kg), median (range)	61	61.5
	(35-103)	(35-94)

BMI (kg/m^2^), median (range)	21.2	21.6
	(16.5-35.6)	(15.6-32.5)

CFS duration (months), median (range)	18.5	31
	(9-72)	(12-83)

Follow-up time (months), median (range)	n.a.	8
		(3-17)

BMI = Body mass index; CFS = chronic fatigue syndrome

At the time of the first visit, patients had high Fatigue severity score, and also a high score on items addressing other CFS symptoms, such as non-relieving rest, concentration problems and post-exertional malaise (Table 2). Also, they reported symptoms of orthostatic intolerance such as lightheadedness, as reflected in the ASP score. However, symptoms such as sore throat and enlarged lymphatic nodes, which both are criteria in the Fukuda definition of CFS [17], were uncommon among our patients.

**Table 2:** Questionnaire results.

	*First visit* *(n = 30-38)^§^*	*Second visit (n = 37-38)^§^*	p-value*
*Fatigue severity score^†^*	4.7	3.9	< 0.001
	(2.9-5.0)	(1.0-5.0)	

*Additional symptoms, score^‡^*			

Non-relieving rest	3.8	3.0	0.005
	(1.0-5.0)	(1.0-5.0)	

Concentration problems	3.7	3.2	0.022
	(1.0-5.0)	(1.0-5.0)	

Post-exertional malaise	3.6	2.6	< 0.001
	(1.0-5.0)	(1.0-5.0)	

Sensitivity towards sounds	3.1	2.6	0.110
	(1.0-5.0)	(1.0-5.0)	

Problems of getting asleep	2.9	3.1	0.621
	(1.0-5.0)	(1.0-5.0)	

Headache	2.8	2.7	0.562
	(1.0-5.0)	(1.0-5.0)	

Muscular pain	2.7	2.2	0.026
	(1.0-5.0)	(1.0-5.0)	

Problems of memory	2.7	2.6	0.715
	(1.0-5.0)	(1.0-5.0)	

Nausea	2.4	2.2	0.267
	(1.0-5.0)	(1.0-4.0)	

Joint pain	2.4	2.1	0.252
	(1.0-5.0)	(1.0-5.0)	

Sore throat	1.7	1.9	0.931
	(1.0-5.0)	(1.0-4.0)	

Enlarged lymphatic nodes	1.5	1.3	0.334
	(1.0-5.0)	(1.0-3.0)	

*Autonomic score^a^*	12.7	12.2	0.436
	(8-21)	(6-22)	

*Functional impairment score^b^*	3.4	2.8	0.003
	(2.0-5.0)	(1.0-5.0)	

Median (range)

^§ ^n varies due to different response rates. * Wilcoxon's signed rank test, 2-sided. ^† ^The average of 7 items, each having a 1-5 Likert scale. ^‡ ^Each single item was scored on a 1-5 Likert scale. ^a ^The sum of 22 dichotomized items addressing different autonomic symptoms; total range is from 0 to 22. ^b ^The average of 3 items, each having a 1-5 Likert scale

During the time span from the first to the second visit, 27 of the patients had tried one or more therapeutic interventions, such as graded exercise therapy, cognitive behavioral therapy and drugs (propranolol, fludrocortisone). Treatment intensity and duration varied considerably; thus, estimating effect from each intervention is not feasible.

At the second visit, there was a significant improvement of mean Fatigue severity score (from 4.7 to 3.9, p < 0.001) (Table 2). On an individual basis, such improvement was experienced by 22 (58%) of the patients, whereas 8 (21%) had unchanged Fatigue severity score and 8 (21%) had worsened score. Also, the mean score of the three most prevalent additional symptoms (non-relieving rest, concentration problems and post-exertional malaise) improved significantly as compared to the first visit, as did the Functional impairment score. Furthermore, there was a significant improvement of muscular pain.

At the time of the first visit, patients had high HR, blood pressure, TPRI and LF/HF at baseline and a strong increase in these variables upon tilt. At the second visit, baseline HR, blood pressure, TPRI and LF/HF were significantly lower and the increase in HR, MBP, DBP and TPRI during tilt were significantly less pronounced as compared to the first visit (Table 3). Thus, most of the hemodynamic variables at the second visit were closer to values observed in healthy controls [6,8].

**Table 3:** Hemodynamic variables at supine rest and during a 20° head-up tilt-test.

	*CFS-patients*			*Healthy controls (n = 33)^†^*
	***First visit*** ***(n = 38)***	***Second visit (n = 37)***	p-value*	

*Baseline*				

HR (beats/min)	72.6	67.5	0.033	68.3
	(68.9-76.9)	(63.5-74.0)		(65.5-72.4)

SBP (mm Hg)	108.3	98.9	< 0.001	111.1
	(105.2-116.1)	(95.3-101.9)		(107.9-116.0)

MBP (mm Hg)	80.4	74.0	< 0.001	79.8
	(77.1-84.8)	(70.5-75.6)		(77.2-81.7)

DBP (mm Hg)	68.1	59.3	< 0.001	64.6
	(64.9-73.1)	(57.5-63.5)		(62.8-65.9)

TPRI (mm Hg/l/min/m^2^)	8.4	7.2	0.001	7.4
	(7.5-9.4)	(6.9-8.2)		(6.9-7.7)

LF/HF	0.6	0.4	0.017	0.6
	(0.4-0.9)	(0.4-0.6)		(0.4-1.0)

*Response (Tilt--Baseline)*				

Δ HR (beats/min)	5.4	2.3	0.030	0.9
	(2.5-6.5)	(1.0-3.0)		(-0.2-2.2)

Δ SBP (mm Hg)	2.1	1.8	0.531	-0.9
	(0.9-3.8)	(-0.2-4.0)		(-1.8-0.9)

Δ MBP (mm Hg)	4.3	1.6	0.001	0.3
	(3.2-6.7)	(0.5-3.3)		(-1.6-1.7)

Δ DBP (mm Hg)	4.1	1.6	0.001	0.4
	(3.6-5.8)	(0.3-2.8)		(-1.5-2.5)

Δ TPRI (mm Hg/l/min/m^2^)	1.0	0.7	< 0.001	0.2
	(0.7-1.6)	(0.3-0.8)		(-0.02-0.5)

Δ LF/HF	0.3	0.2	0.187	-0.03
	(0.1-0.5)	(0.1-0.3)		(-0.2-0.1)

Median (confidence intervals)

* Wilcoxon's signed rank test, 2-sided, between first and second visits for the CFS patients. ^†^Although not the primary scope of this article, reference values from healthy control subjects are displayed for clarity. Details concerning this reference material have been described elsewhere [8]. In order to avoid methodological problems of multiple comparisons, statistical tests between patients and control subjects have not been carried out

HR = heart rate; SBP = systolic blood pressure; MBP = mean arterial blood pressure; DBP = diastolic blood pressure; TPRI = total peripheral resistance index; LF/HF = Ratio of low-frequency to high frequency power of heart rate variability

The change in Fatigue severity score from the first to the second visit was significantly correlated with change in Autonomic score (Table 4). Also, change in Functional impairment score was significantly correlated with change in Fatigue severity score.

**Table 4:** Correlation* between changes (first to second visit) of selected variables

	**Change in Fatigue severity score**^**†**^		Change in Autonomic **score**^**a**^	
	***τ***	***p-value^§^***	***τ***	***p-value^§^***

Change in Fatigue severity score^†^	n.a	n.a	0.27	0.044

Change in Functional impairment score^b^	0.36	0.003	0.14	0.291

* Kendall rank correlation. § Kendall's tau test, 2-sided. ^† ^The average of 7 items, each having a 1-5 Likert scale. ^a ^The sum of 22 dichotomized items addressing different autonomic symptoms; total range is from 0 to 22. ^b ^The average of 3 items, each having a 1-5 Likert scale. τ = tau (correlation coefficient)

## Discussion

The most important findings of this follow-up study are:

1) An improvement of self-reported fatigue and other common CFS symptoms.

2) An improvement of hemodynamic responses during HUT.

3) Correlations between changes in autonomic symptom score, fatigue severity score and functional score.

### Improvement of fatigue and other symptoms

FSS is a commonly used, validated tool for measuring fatigue in various diseases, and has a documented ability to detect change over time [34,35]. A highly significant improvement as measured by FSS therefore clearly displays the clinical improvement of our adolescent patient group. Importantly, this improvement was accompanied by improvement of other symptoms contributing significantly to the patients' disability, as well as a significant improvement of self-reported functional level.

### Improvement of hemodynamic variables during HUT

Altered autonomic nervous activity characterized by enhanced sympathetic cardiovascular control has been documented by several CFS researchers [2-10], and might constitute a key element of CFS pathophysiology [11]. Indeed, previous experiments from our institution applying HUT and related techniques did find increased HR, blood pressure, TPRI and LF/HF at rest, and a stronger increase in these variables upon orthostatic challenge in CFS patients as compared to healthy controls [6-9]. In this study, the haemodynamics among CFS patients at the first visit were similar to previous observations. However, at the second visit, CFS patients had significantly lower baseline values for all measured hemodynamic variables as compared to the fist visit, indicating a decrease in sympathetic cardiovascular influence at rest. Furthermore, the changes in hemodynamic variables during tilt were smaller, indicating a less pronounced sympathetic cardiovascular influence during orthostatic challenge. Thus, at the second visit, the HUT results among CFS patients were closer to what has previously been reported among healthy adolescents [6,8].

### Correlation between changes in autonomic score, fatigue score and function score

The concomitant improvement of symptoms, HUT-responses and self-reported functional abilities suggests a possible causal relation between these variables, in line with recent theories advocating autonomic abnormalities as an important part of CFS pathophysiology [11]. The significant correlation between changes in autonomic symptoms, fatigue and functional impairment further strengthens this notion. However, we failed to demonstrate a statistically significant correlation between changes in questionnaire variables and changes in HUT-variables. This might be due to methodological constraints (few participants, no normal distribution of variables). In addition, the relation between symptoms and certain pathophysiological characteristics might be complex and non-linear, as has recently been suggested for the subtle alterations of the hypothalamus-pituitary-adrenal system in CFS [36].

This study was not designed for assessment of causal relations; for instance, autonomic abnormalities might cause fatigue, fatigue might give rise to autonomic abnormalities, or they might both be the product of a third variable. Further studies should focus on these questions. If they confirm a pathophysiological role of altered cardiovascular autonomic control in CFS, this phenomenon might constitute a possible target for therapeutic interventions, e.g. with pharmaceuticals attenuating sympathetic nervous activity. In addition, the responses to HUT might emerge as a possible biomarker of the disease processes.

### Study limitations

A challenge regarding CFS research is the diversity in case definition and inclusion criteria. This study applied a modification of the Fukuda-definition, and our results cannot be readily compared with studies adhering strictly to this definition. Of note, our patients had remarkably low scores on some of the additive criteria of the Fukuda definition of CFS, in particular sore throat and tender lymph nodes [17]. This adds to the ongoing discussion of the validity of this definition [18]; at least, our findings indicate that a strict application of these criteria may correspond to a small fraction of all adolescents with long-lasting, unexplained and disabling fatigue. Thus, omission of these additive criteria, as applied in this study and advocated by the guidelines from the Royal College of Paediatrics and Child Health [1] and the National Institute for Health and Clinical Excellence [20], seems to increase the ecological validity and improves the generalizability of the results towards routine clinical care

All participants in this study were recruited from a national referral center, possibly causing a selection bias: those being less severely affected from CFS might not have come to our attention. This might shift our results towards worse outcome regarding the entire adolescent CFS population. On the other hand, no one was permanently bed-ridden; thus, the most severely affected patients did not participate in this study.

The questionnaire has not been formally validated. However, its major components, such as the Fatigue Severity Scale and The Autonomic Symptom Profile, are validated instruments; the translated and slightly adjusted versions of these instruments have proven feasible in previous studies from our institution [7,8].

At the first visit, patients had fasted overnight and all tests were performed between 8 and 11 a.m. The same strict regime did not apply to HUT at the second visit. This might bias our data due to diurnal variation in HUT responses. However, whereas a recent study by Brewster and co-workers demonstrated higher HR in the morning in a group of patients with the postural orthostatic tachycardia syndrome, no diurnal variations were found for blood pressure [37].

Only 37 patients were included, but based on experiences from earlier studies at our institution, we had reason to assume that a sample of more than 30 patients would be sufficient to discover significant and clinically interesting changes in a follow-up study.

We cannot rule out that the changes in hemodynamic responses from the first to the second visit could be partly or totally explained from habituation; i.e., that the participants were less anxious and uncomfortable at the second visit. However, this possibility does not explain the improvement of symptoms. Also, changes in hemodynamic responses could be explained from natural pubertal development of the patients during the same period. However, in linear regression analyses, the number of months between the first and the second visit was not significantly related to changes in HUT variables (results not shown).

Finally, some of the patients received different kind of treatment between the first and second visit. The design of the study does not allow detailed analyses of the relation between treatments and clinical improvements. Indeed, further studies should explore these relations within a strictly controlled design.

### Concluding remarks

In conclusion, our study shows an improvement over time in self-reported fatigue and other common CFS- symptoms, in line with several other studies. Also, we have demonstrated a concomitant improvement of hemodynamic variables and functional abilities, supporting a theory of autonomic abnormalities as an important part of CFS pathophysiology. The results call for further investigation regarding autonomic control in CFS, research that will, hopefully, reveal pathophysiological mechanisms that might open new therapeutic options.

## Competing interests

The authors declare that they have no competing interests.

## Authors' contributions

DS, HH and IBH carried out the clinical work. DS drafted and completed the manuscript. ET contributed on study design and manuscript corrections. VBW conceived of the study, and contributed to statistical analyses and manuscript corrections. All authors read and approved the final manuscript.
